# The Effects of Different Miniscrew Thread Designs and Force Directions on Stress Distribution by 3-dimensional Finite Element Analysis

**Published:** 2015-12

**Authors:** Hamidreza Fattahi, Shabnam Ajami, Ali Nabavizadeh Rafsanjani

**Affiliations:** a Orthodontic Research Center, Dept. of Orthodontics, School of Dentistry, Shiraz University of Medical Sciences, Shiraz, Iran.; b Dept. of Orthodontics, School of Dentistry, Rafsanjan University of Medical Sciences, Rafsanjan, Iran.

**Keywords:** Miniscrew, Thread, Finite Element Analysis, Force Direction

## Abstract

**Statement of the Problem:**

The use of miniscrew as an absolute anchorage device in clinical orthodontics is growing increasingly. Many attempts have been made to reduce the size, to improve the design, and to increase the stability of miniscrew.

**Purpose:**

The purpose of this study was to determine the effects of different thread shapes and force directions of orthodontic miniscrew on stress distribution in the supporting bone structure.

**Materials and Method:**

A three-dimensional finite element analysis was used. A 200-cN force in three angles (0°, 45°, and 90°) was applied on the head of the miniscrew. The stress distribution between twelve thread shapes was investigated as categorized in four main groups; buttress, reverse buttress, square, and V-shape.

**Results:**

Stress distribution was not significantly different among different thread shapes. The maximum amount of bone stress at force angles 0°, 45°, and 90° were 38.90, 30.57 and 6.62 MPa, respectively. Analyzing the von Mises stress values showed that in all models, the maximum stress was concentrated on the lowest diameter of the shank, especially the part that was in the soft tissue and cervical cortical bone regions.

**Conclusion:**

There was no relation between thread shapes and von Mises stress distribution in the bone; however, different force angles could affect the von Mises stress in the bone and miniscrew.

## Introduction

Orthodontic miniscrews have revolutionized orthodontic treatment plans. Nowadays, the use of miniscrew as an absolute anchorage in clinical orthodontics is growing increasingly. Some reasons for this growth include easy insertion and removal of the miniscrew without irreversible changes,[1] immediate loading,[2] low cost of the instruments, and shorter duration of the treatment.[3-4]

One of the requirements of immediate loading is primary stability[5-7] which is influenced by several factors including the design of the miniscrew, implant size, insertion angle, insertion torque, force angle, and the amount of applied force.[8] Design of the miniscrew is characterized by some factors such as the length and diameter of the miniscrew, thread shape, pitch, and depth.[9] Different thread shapes have been introduced, the basic forms of which are square, V-shape, buttress and reverse buttress.[8] Attempts to maximize the stability while minimizing the placement torque has led to the development of smaller miniscrews, which would broaden their clinical use.

Many studies on the design of orthodontic miniscrew and few on orthodontic miniscrew thread shapes have been conducted.[5, 8-9] Gracco *et al.* conducted an *in vitro* study to evaluate the effect of thread shapes on the pullout strength of the miniscrews. They designed four types of thread named as buttress, 75 joint profiles, rounded, and trapezoidal. They concluded that the thread design influenced the resistance to pullout and consequently the primary stability of orthodontic miniscrews.[7] Migliorati *et al.* used thread shape factor to determine the relationships between geometrical characteristics and mechanical properties of the temporary anchorage devices. Their results showed that maximum insertion torque and load values of the pull-out test were statistically related to the depth and shape of the thread of the screw.[11] Duaibis *et al.* showed that different thread shapes had no effect on the stresses around the cortical bone.[12] However, some studies, carried out on dental implants, have indicated that a key factor for the success or failure of dental implants would be the type and the amount of the bone stress.[8, 13] Kong *et al.* designed a finite element study to determine the optimal thread shape for an experimental cylinder dental implant. Twelve 3D models of dental implants with different thread shapes were investigated. They concluded that some of the thread shapes had better stress distribution.[14] Liu *et al.* showed that the direction of orthodontic forces had no significant effect on the cortical bone stress. However some other studies did not support this finding.[15-16] So there is a controversy over the impact of thread shapes on the bone stress and stability. Regarding this gap in literature, we decided to carry out a study to determine the effect of different thread shapes and force angles on stress distribution of bone and miniscrew. In our study, finite element analysis was used to evaluate the effect of different miniscrew thread shapes and load angles on stress distribution around miniscrew and supporting bone.

## Materials and Method

**Figure 1:**
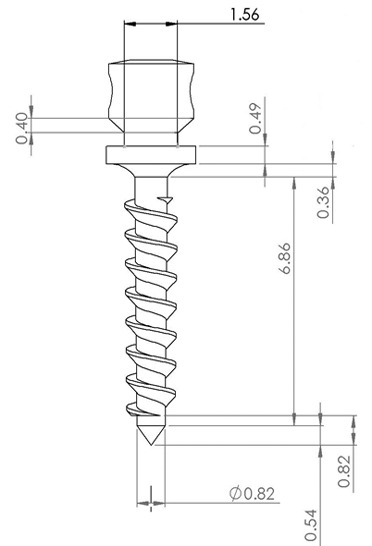
Detailed dimensions of the miniscrew

A three dimensional (3D) geometric model of a miniscrew as a bone anchorage was created with the computer aided design software SolidWorks2013 (Figure 1).

Cortical and cancellous bones were modeled. A cuboid of 20mm long, 20mm wide and 10mm thick was modeled, considering the upper 1.5 mm as soft tissue, the upper 2 mm was considered as cortical bone and the rest as cancellous bone. The material properties of the elements in finite element are shown in Table 1.[10]

                                                  

**Table 1:** Properties of the material used in this investigation

	**Young’s modulus** **(GPa)**	**Poisson’s** **ratio**	**Reference**
Pure titanium	110	0.33	10
Cortical bone	14.7	0.30	10
Cancellous bone	1.3	0.30	10

The characteristics of the miniscrew based on the model designed by Singh *et al.* using microscope tool mark were as fallowing;[9] the total length of miniscrew was 10.62 mm and the length of the threaded shank was 6.84 mm. The tip was 0.54 mm long. Shank of the miniscrew had tapering with the diameter of 0.95mm in the largest part without considering the thread width and 0.82 mm in the smallest diameter. The part of miniscrew outside the bone was 3.22 mm. The largest diameter of the head was 2.48 mm and the smallest part was 1.56 mm. The thread pitch was considered 0.8 mm and was arranged on the shank in a spiral pattern. The miniscrew was inserted at the right angle in the bone.

**Figure 2:**
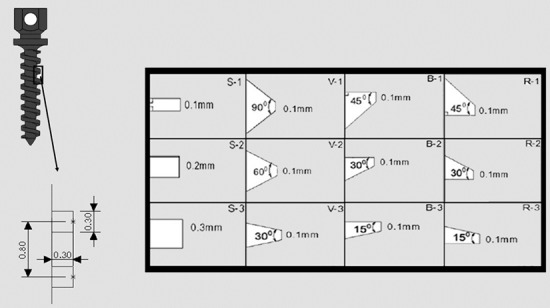
Schematic representation of the miniscrew thread shapes:  (S=square; V=V-shaped; B=buttress; R=reverse buttress)

For better understanding of the stress distribution, the twelve different thread configurations were categorized in four main groups: buttress (B-1, B-2, and B-3), reverse buttress (R-1, R-2, and R-3), square (S-1, S-2 and S-3), and V-shape (V-1, V-2 and V-3). Thread depth (the distance between shank and the thread tip) was 0.3mm in all configurations. The difference between buttress, reverse buttress, and V-shape groups was the angle between the two wings of the thread. The square group was divided by the thread width. The details of these designs are showed in Figure 2.

All the materials in this modeling were considered homogeneous which means the elastic properties were all the same at all points in the material, isotropic which means the same elastic properties existed in all directions at any point of the material, and linearly elastic which was an acceptable assumption due to the small deformation occurred during loading. Hence, there was a linear relation between stress and strain. Bone and miniscrew had a finite slip and the friction coefficient equal to 0.2, as suggested by Lombardo *et al.*[17] Finite element model was constructed and automatically meshed with 10 node tetrahedral solid elements (solid 186 and solid 187) by using ANSYS Workbench Version 14 (Southpointe; 275 Technology Drive, Canonsburg PA 15317, USA).In all models, the number of elements varied between 850000 to 1200000 due to the difference in the thread shapes of the miniscrew. In all cases, the maximum skewness of the worst element was <0.9 and minimum orthogonal quality of the worst element was >0.15.

For stimulating the pull force on the head of miniscrew, a 200-cN force was applied on the head of the miniscrew in 3 directions (0°, 45° and 90°). The 90° force angle was parallel to the cortical surface, perpendicular to the long axis of the miniscrew, and the 0° force angle was along the long axis of the miniscrew in positive direction of y axis (extrusive). The 45° force angle was between 0° and 90° force angles. Finally, 36 different modes were simulated. The effects of miniscrew thread design and different force directions (0°, 45°, and 90°) on the stress distribution were investigated.

The results of finite element analysis were expressed as stress distribution in the structures. The stresses included tensile, compressive and shear which can interpret as von Mises stress or equivalent stress. Von Mises stress is widely used by designers to make sure whether their design withstands the given loaded condition.[8, 18] The calculated numerical data were shifted into color band diagram for better understanding of the mechanical phenomena in models. The stress values were indicated in mega Pascal (MPa) or Newton per square millimeter. Deformation due to horizontal loading predominantly occurred in the x-axis direction and the values were reported in millimeters.

## Results

**Bone stress distribution**

**Figure 3:**
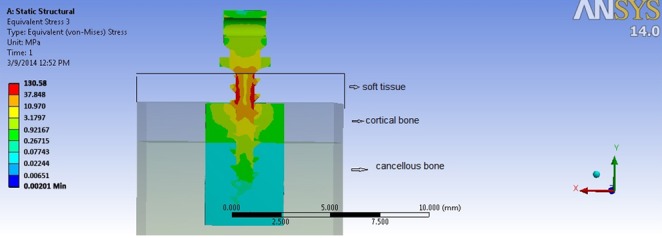
Stress distribution in surrounding bone

Generally, the amount of stress for all force directions (0°, 45°, and 90°) and in all thread shapes was greater in the cortical bone than the cancellous bone. The greatest amount of stress in cortical bone was at the entrance of the miniscrew to the bone; the stress levels reduced gradually toward the tip of the miniscrew (Figure 3).

At the 0° force angle, the peak von Mises stress of the cortical bone ranged between 35.87 to 38.90 MPa with the minimum amount for the buttress 1(B-1) and reverse buttress 2(R-2) thread shapes and the maximum amount for buttress 3 (B-3) thread shape. However, when the angle was increased by 45 degrees, the peak von Mises stress decreased to 26.5-30.57 MPa, the lowest amount of which was observed in reverse buttress 1(B-1) and the greatest in square 3(S-3) and V-shape 3(V-3) thread shapes. At 90° force angle, the peak von Mises stress declined significantly and the lowest stress was related to V-shape 1(V-1) (5.74 MPa) and the highest was related to buttress 3 (B-3) thread shape (6.62 MPa). (Table 2) Table 3 shows the maximum shear stress.

**Table 2:** Maximum cortical equivalent stress under 200-cN force (MPa)

	**B1**	**B2**	**B3**	**R1**	**R2**	**R3**	**S1**	**S2**	**S3**	**V1**	**V2**	**V3**
Force angle 0°	35.87	36.38	38.90	36.58	35.87	36.81	36.87	36.40	37.26	36.56	38.45	37.31
Force angle 45°	26.79	29.06	27.55	26.50	30.37	29.11	29.42	29.58	30.52	28.07	30.23	30.57
Force angle 90°	6.02	6.00	6.62	6.12	6.51	6.05	6.43	6.12	6.35	5.74	6.41	6.24

B: Buttress thread shape, R: Reverse buttress thread shape, S: Square thread shape, V: V-shape thread shape

**Table 3:** Maximum cortical shear stress under 200-cN force ( MPa)

	**B1**	**B2**	**B3**	**R1**	**R2**	**R3**	**S1**	**S2**	**S3**	**V1**	**V2**	**V3**
Force angle 0°	18.736	19.048	20.4925	19.067	18.716	19.189	19.232	18.990	19.603	19.147	20.292	19.522
Force angle 45°	13.955	15.210	14.325	13.722	16.094	15.307	15.447	15.587	16.091	14.712	15.958	16.136
Force angle 90°	1.160	1.155	1.292	1.181	1.267	1.1616	1.247	1.1867	1.230	1.0963	1.248	1.206

B: Buttress thread shape, R: Reverse buttress thread shape, S: Square thread shape, V: V-shape thread shape

**Miniscrew stress distribution**

**Figure 4:**
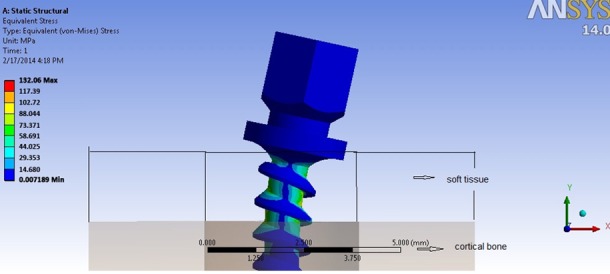
Stress distribution along a miniscrew shank

The peak von Mises stress in the miniscrew was at the smallest diameter of the shank, especially in the part inserted in the soft tissue. Generally, toward the tip of miniscrew, this value was decreased. For the part of the miniscrew located within the cortical bone, the greatest amount of stress was in the narrowest part of the shank (Figure 4).

Applying the 0° force angle, the peak von Mises stress of the miniscrew was ranged from 122.62MPa to 132.06 MPa, the lowest and greatest amount were for V-shape 1(V-1) thread shape and reverse buttress 1(R-1) thread shape, respectively. However, when the angle of force increased to 45^o^, the peak von Mises stress decreased with lowest amount for square 1(S-1) thread shape (93.10 MPa) and the greatest for reverse buttress 3(R-3) thread shape (108.73 MPa). At 90° force angle, the peak von Mises stress declined significantly. The range was from 20.78 MPa to 22.85 MPa, with the lowest amount for V-shape 1 thread shape and the largest for reverse buttress 3 (R-3) thread shapes. (Table 4)

**Table 4:** Maximum miniscrew equivalent stress under 200-cN force (MPa)

	**B1**	**B2**	**B3**	**R1**	**R2**	**R3**	**S1**	**S2**	**S3**	**V1**	**V2**	**V3**
Force angle 0°	130.58	131.66	131.19	132.06	130.58	132.00	122.85	122.87	125.04	122.62	126.91	124.50
Force angle 45°	107.54	108.40	104.07	108.63	105.06	108.72	93.10	99.01	103.01	100.60	100.15	99.91
Force angle 90°	22.56	22.78	22.58	22.64	22.57	22.85	21.49	21.27	21.54	20.78	21.43	21.84

B: Buttress thread shape, R: Reverse buttress thread shape, S: Square thread shape, V: V-shape thread shape

**Deformation**

**Figure 5:**
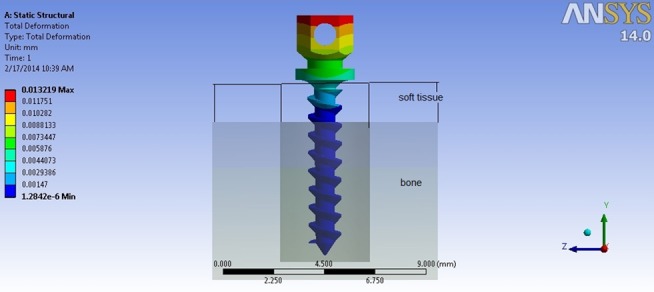
Deformation patterns in a miniscrew model under horizontal loading

The maximum deformation in all groups was at the head of the miniscrew, whereas to the tip of the miniscrew, this value was reduced. (Figure 5) With the 0° force of angle loading, the maximum value was for square 1(S-1) thread shape (0.0116 mm) and the minimum was for V-shape 2 (V-2) thread shape (0.0154 mm). At the 45° force angle, the result showed the range was between 0.0070 mm and 0.0109 mm. The largest value was for square 1(S-1) thread shape and the minimum was for V- shape 1(V-1) thread shape (Table 5).

**Table 5:** Maximum miniscrew deformation under 200-cN force (µm )

	**B1**	**B2**	**B3**	**R1**	**R2**	**R3**	**S1**	**S2**	**S3**	**V1**	**V2**	**V3**
Force angle 0°	13.225	14.151	14.873	12.360	13.653	14.611	15.425	14.172	13.377	14.836	11.621	13.364
Force angle 45°	9376	10.026	10.529	8.760	9.681	1.0349	10.912	10.044	8.915	7.064	8.674	9.929
Force angle 90°	0.182	0.186	0.187	0.187	0.189	0.189	0.187	0.191	0.188	0.173	0.185	0.188

B: Buttress thread shape, R: Reverse buttress thread shape, S: Square thread shape, V: V-shape thread shape

## Discussion

This study was conducted to evaluate the effects of various thread shapes and force directions on the stress distribution in different parts engaged in the process of loading.

The purpose of having threads on the miniscrew is to enhance the initial contact and surface area, which will optimize the stress distribution in the contact areas between the bone and the miniscrew.[8, 19] Various forms of screw thread can have different impacts on the initial stability. Some of the thread forms introduced for dental implants are the square, V-shape, and buttress. The V-shape thread, also known as fixture, is primarily used for fixing metal parts together, not to transfer the load. On the other hand, the buttress thread is resistant to pullout force and the square threads provide an appropriate surface area for transmitting compressive and intrusive forces.[20] Applying the 200-cN force with 45° and 90° angles showed that the greatest amount of stress was in the cortical bone which was significantly higher than that of the cancellous bone. This result might be due to the different modulus of elasticity between these two types of bone. The result was similar to the previous studies.[21-22] However in this study; the thickness of soft tissue was also considered while it was not investigated in the study of Singh *et al.*[9] This might be attributed to the effect of the lever arm of the miniscrew since the bending moment increased with the elongation of the lever arm. Excessive bone stress might cause local bone resorption. Other reasons for the difference between the amounts of von Mises stress in various studies are different mesh design and size, as well as the shape and diameter of the screws, different Young’s modulus,[23] and deliberation of friction.

In this study, like most other studies, the maximum von Mises stress levels with 200-cN horizontal force was less than the yield strength of cortical bone (133 MPa).[10] So, it can be concluded that all of the thread designs could be safe as a bone anchorage device when applying 200-cN force.

The maximum shear stress criterion, also known as Tresca's criterion, is often used to predict the yield strength of ductile materials. Since the maximum shear stress criterion is more conservative than the von Mises, the results obtained by von Mises are larger than the results obtained by Tresca; therefore, we only discussed the von Mises stress.

As can be noted, various shapes of threads showed different behaviors at different force directions. In applying 0° force angle, buttress 1(B-1) and reverse buttress 2(R-2) exerted the least amount of stress to the bone; while, the buttress 3 did the greatest. With 90° force angle, V-shape 1(V-1) thread showed the least amount of stress to the bone and buttress 3 showed the greatest. This can be explained by the fact that in the V-1, the two sides of the thread altogether made a 90° angle which had less integration than the other thread shapes and acted as a ramp in facing tangent force to the long axis of the miniscrew. This thread shape had also more surface area than the other types; therefore, under the same loading condition it transmitted less stress to the bone. Buttress 3 (B-3) threads produced the highest amount of stress with such force direction, this superiority over V-shape and reverse buttress group can be explained by the fact that the latter two groups had a ramp relative to the force direction .Moreover, better lock mode was observed in the square and buttress groups. Regarding these results at the 0° force angle, there was no significant superiority in any types of the thread shapes tested in this study. This result was in contrast with Gracco *et al.’s* findings; probably because of the different types of the tests performed.[7] They used pullout test to assess the initial stability, and found that the thread design influenced the resistance to pullout and the primary stability of orthodontic miniscrews. It should be mentioned that in the pullout test, the applied force was parallel to the long axis of the miniscrew; while, the orthodontic force in the mouth is mostly applied perpendicularly to the miniscrew, so stress distribution is quite different under these 2 loading conditions. Duaibis *et al.* showed that the thread shapes could not generate different patterns of stress distribution in the surrounding bone.[12] Three designs of miniscrews were included in their study including no thread, asymmetrical triangle and symmetrical triangle; whereas, we examined 12 different thread shapes. The amount of bone stress and also the difference in stress levels among different thread shapes were higher in our study, and the results were similar to the studies performed by Eraslan and İnan, Kong *et al.*, and Geng *et al.* on dental implants.[8, 14, 24] It should be noted that the type of force loaded on dental implants is usually compressive and is different from the load on orthodontic miniscrews, which is mainly torsion or tangential.

According to the results of this study, reducing the force direction from 90° to 45° and from 45° to 0° led to decreased amount of bone stress. This can be attributed to the fact that at 90ᵒ the whole force was applied in the horizontal direction, but at 45° the force was divided into two main components, namely horizontal and vertical. As mentioned in the study by Liu *et al.*, a horizontal load would induce much more stress than a vertical load.[15] The contribution of each component is 141cN; thus, the horizontal force of

141cN logically provides less stress than pure horizontal force with the magnitude of 200 cN at 90°.

According to the findings, under the same loading conditions, different shapes of miniscrew thread did not revealed a significant difference in the amount of maximum von Mises stress. It was lower than the yield strength of pure titanium for all thread shapes and force directions. The highest amount of stress was seen in the area with the smallest diameter just above of the entrance of miniscrew to the cortical bone. Therefore, the risk of failure is higher in this area. This can be justified with the second moment of inertia of a cylinder that shows the peak stress is inversely proportional to the third power of the diameter. These findings were different from the result achieved by Singh *et al.*[9] due to the fact that they did not consider a space for the soft tissue and reported the maximum stress to be located in the neck of miniscrew. Meanwhile, our result was similar to the results yielded by the study of Liu *et al.*[15] According to these results, it can be suggested that the part of the miniscrew which is inside the soft tissue should have a larger diameter relative to the portion that is located inside the cortical bone. Simultaneously, the thread width of the soft tissue area should be reduced compared to those in contact with the bone.

The thread shapes did not have a significant impact on the miniscrew deflection under the horizontal loading. This value decreased with the reduction of the angle of force because the amount of horizontal force was reduced as previously described.

Like other finite element studies, this study had some limitations in the simulation.[8-9,12,15,22-23] The structures in the models were assumed to be linear, homogeneous, and isotropic; while, real bone is neither homogeneous nor isotropic,[12, 25] but we used these assumptions for simplicity and to compensate the lack of information on the bone behavior. The geometry of the bone block was simplified to a rectangular block instead of a jaw section. The soft tissue was not simulated; although its thickness was deliberated. We assumed that the friction coefficient between the miniscrew and bone was 0.2; it might be different for cortical and cancellous bones. Since the thread shape might affect the insertion of miniscrew in the bone, further studies on this subject are recommended.

## Conclusion

Considering the limitations of this study, two conclusions can be drawn: first, different thread shapes did not affect the pattern of distribution and the amount of von Mises stress; second, different force angles affected von Mises stress. 
